# Prevention preferable to treatment: 3 case reports of patients experiencing right-sided heart failure after Ebstein anomaly correction

**DOI:** 10.1097/MD.0000000000005627

**Published:** 2017-01-10

**Authors:** Ming Luo, Jing Lin, Zhen Qin, Lei Du

**Affiliations:** Department of Anesthesiology and Translational Neuroscience Center, West China Hospital, Sichuan University, Chengdu, China.

**Keywords:** arginine vasopressin, AVP, cardiopulmonary bypass, continuous renal replacement therapy, CPB, CRRT, ECMO, extracorporeal membrane oxygenation, ICU, intensive care unit, MAP, mean arterial pressure, TEE, transesophageal echocardiography

## Abstract

**Rationale:**

Ebstein anomaly is a common congenital heart disease that may induce severe tricuspid regurgitation and dilation of the “atrialized” portion of the right ventricle. Patients who undergo surgery to correct Ebstein anomaly are at high risk of postoperative right-sided heart failure, yet little is known about what pre-, peri-, or postoperative procedures may help reduce this risk.

**Patient concerns:**

Here, we describe 3 cases of adults with Ebstein anomaly who underwent corrective surgery and in whom right-sided heart failure occurred with severe tricuspid regurgitation detected by transesophageal echocardiography.

**Diagnoses:**

Ebstein anomaly.

**Intervention:**

Various approaches were applied to prevent right heart failure: perioperative control of atrial and ventricle arrhythmia, protection of myocardium, reduction of right-side cardiac workload after cardiopulmonary bypass, and mechanical support for right heart.

**Outcomes:**

One of the 3 patients died, another experienced kidney failure despite postoperative support on extracorporeal membrane oxygenation, and the third patient survived without complications.

**Lessons:**

Our case series suggests that surgical prognosis can be improved through aggressive preoperative treatment, vasoactive and anti-arrhythmia medications, and comprehensive measures designed to reduce myocardial injury, prevent myocardial edema, and reduce pre- and afterload on the right ventricle.

## 1 Introduction

In patients with Ebstein anomaly, severe tricuspid regurgitation significantly increases preload on the right ventricle. In early disease, the body attempts to maintain cardiac output of the right ventricle by dilating it and increasing cardiac contractility.^[1]^ In advanced disease, tricuspid regurgitation severely expands both the right atrium and right ventricle, ultimately resulting in right ventricle failure.^[2]^ Patient quality of life may be improved through tricuspid valve repair or replacement under cardiopulmonary bypass (CPB). However, such surgery and the associated ischemia-reperfusion injury can increase the risk of right-sided heart failure. Such failure occurs in up to 13% of patients following correction of Ebstein anomaly, and in-hospital mortality ranges from 5% to 16.6%.^[3–5]^

It is possible that studying the physiological alterations contributing to right ventricle failure in patients with Ebstein anomaly can guide efforts to avoid such failure and thereby improve surgical outcomes. Therefore, we reviewed 3 cases of surgical patients with Ebstein anomaly and right ventricle dysfunction.

Informed consent to be included in this report was obtained from the patients or their guardians.

## 2 Case 1

**Figure 1:**
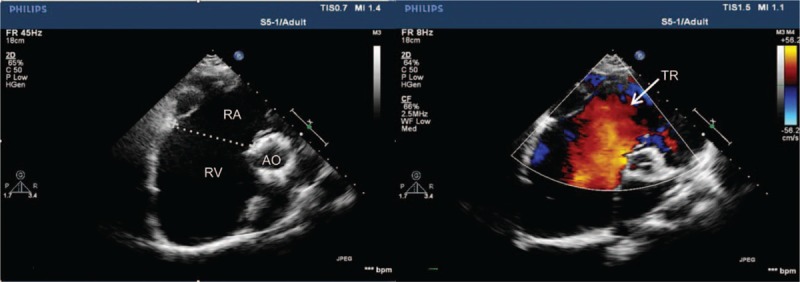
Preoperative echocardiography showing enlargement of the right chamber and severe tricuspid regurgitation. These images come from Case 1 and are representative of all 3 cases in this series. RA = right atrium, RV = right ventricle, TR = tricuspid regurgitation.

A 24-year-old female weighing 50 kg was admitted to our hospital in November 2012 because of a cardiac murmur detected during routine testing before her wedding. She had a history of recurrent respiratory tract infection as a child; during these infections, she often experienced palpitations, cyanosis, and dyspnea, which resolved with rest. These symptoms disappeared after atrial septal defect repair and tricuspid valve repair when she was 9 years old. Echocardiography after the most recent admission showed tricuspid valve hypoplasia with inferiorly displaced septal and posterior leaflets, severe tricuspid regurgitation, substantial dilation of the right atrium and ventricle, reduced systolic function of the right ventricle wall, and a relatively small left atrium and ventricle (Fig. 1).

Atrial tachycardia was recorded by routine ECG and right atrial tachycardia was confirmed during electrophysiology assessment. Then it was corrected by catheter ablation, before she was transferred to the cardiac care unit. One episode of heart block that progressed from first-degree to type II second-degree was recorded, during which heart rate varied from 55 to 65 bpm. The patient did not report dyspnea or fatigue.

On the following day, routine anesthesia was administered involving inhalation of sevoflurane, intravenous infusion of propofol, and intermittent administration of sufentanil, midazolam, and cis-atracurium. Hemodynamics remained stable during anesthesia.

CPB was established by canalizing the right femoral vein and artery after mid-sternal thoracotomy, then primed with Voluven (1000 mL), albumin (20 g), mannitol (100 g), and heparin (30 mg). During the 143-minute cross-clamping period, cold-blood antegrade cardioplegia was administered by infusion at intervals of 25 to 28 minutes. When body temperature stabilized at 32 to 33°C, cone reconstruction of the tricuspid was performed, an epicardial pacemaker was inserted, and the atrial septal defect was preserved. The cross-clamp was removed and the heart beat spontaneously recovered. Supportive CPB was again performed for 35 minutes, after which the patient was weaned off bypass and supported on epinephrine (0.1 mcg/kg/min), milrinone (0.8 mcg/kg/min), and norepinephrine (0.1 mcg/kg/min). The patient's heart rate was 100 to 110 bpm and arterial blood pressure was 90–100/50–60 mm Hg.

CPB lasted for a total of 273 minutes. During that time, 4.5 U of packed red blood cells were transfused, and ultrafiltration was used to keep hematocrit above 20%. Postoperative transesophageal echocardiography (TEE) showed mild tricuspid regurgitation, antegrade flow patency, and an atrial directional shunt.

After the patient arrived in the intensive care unit (ICU), her blood pressure decreased rapidly; it rose to 100/50 mm Hg with infusion of arginine vasopressin (AVP, 0.04 U/min). Another episode of hypotension occurred involving a heart rate of 120 bpm and the alternating presence of sinus and atrial rhythms; the condition resolved with AVP. Hypotension due to recurrent atrial tachycardia occurred again on day 2, but it did not respond even to higher doses of vasoactive and inotropic agents. TEE revealed marked dilation of the right ventricle and poor systolic and diastolic function. Extracorporeal membrane oxygenation (ECMO) was performed via femoral arterial and venous cannulation. Overdrive pacemaker suppression restored normal sinus rhythm, which was maintained by intravenous infusion of amiodarone.

The patient was taken off ECMO on day 3 after achieving hemodynamic stability (mean arterial pressure [MAP], 85 mm Hg) and maintained at an inspired O_2_ fraction (FiO_2_) of 55%; positive end-expiratory pressure (PEEP), 7 cmH_2_O; and Hb, 103.1 to 113.6 g/L. Refractory hypotension occurred 1 hour later, so thoracotomy followed by cannulation of the central aorta and right atrium was performed to reestablish ECMO. The patient underwent an emergency bidirectional Glenn procedure in the operating room, and then was transferred back to the ICU under ECMO assistance. She was discharged on day 4 at the request of her family and died immediately after discharge.

## 3 Case 2

A 61-year-old female weighing 89 kg presented with cyanosis and palpitations that progressively worsened over 4 months. Physical examination showed an enlarged heart, regular heart rate, and blood pressure of 143/88 mm Hg. Echocardiography showed the presence of Ebstein anomaly involving an apical-ward shifting tricuspid valve, severe tricuspid regurgitation, patent foramen ovale (PFO) with bidirectional shunts, and a dilated right ventricle and left atrium. Left ventricle size was normal. Electrocardiography showed complete right bundle branch block.

Anesthesia and CPB proceeded normally. During CPB, cold-blood cardioplegia infusion was administered at 25-minute intervals to protect the myocardium. During aortic cross-clamping lasting 64 minutes and CPB lasting 100 minutes, the tricuspid was replaced, the PFO was closed and a temporary pacemaker was inserted. The cross-clamp was removed and the heart beat spontaneously recovered. No tricuspid regurgitation was detected using postoperative TEE. The patient was weaned off CPB and supported by inotropics (epinephrine, 0.05 mcg/kg/min; milrinone, 0.5 mcg/kg/min; norepinephrine, 0.05 mcg/kg/min).

At 13 hours after surgery, the patient experienced an episode of ventricle tachycardia, during which the heart beat rose to 226 bpm and central venous pressure (CVP) rose to 17 cmH_2_O; subsequently, MAP decreased to 35 mm Hg. Asynchronous defibrillation shock was administered without success, then ECMO was performed using optimal inotropics (epinephrine, 0.18 mcg/kg/min; norepinephrine, 0.24 mcg/kg/min; AVP, 0.07 U/min) to stabilize hemodynamics. Grade 3 acute kidney injury (defined as described)^[6]^ was indicated by a decline in daily urinary output to 545 mL, followed by oliguria and elevated levels of urea nitrogen (9.65 μmol/L), creatinine (203 μmol/L), and uric acid (405 μmol/L). The patient was immediately given continuous renal replacement therapy (CRRT).

When MAP stabilized at 50 to 100 mm Hg and normal sinus rhythm returned at a heart beat of 70 to 110 bpm, the patient was gradually taken off vasoactive agents but kept on ECMO assistance. TEE indicated progressive recovery of right ventricle function. On day 7, the patient was weaned off ECMO using low-dose epinephrine and norepinephine, but she was maintained on CRRT.

The patient underwent tracheotomy on day 12, and she was weaned off the ventilator on day 23 due to recurrent arrhythmia during multiple CRRT. She was discharged from the ICU on day 31 with bedside CRRT. On day 61, she suffered from 1 episode of sudden cardiac arrest. After successful cardiopulmonary resuscitation, she was readmitted to the ICU and extubated 4 days later. She subsequently experienced type I respiratory failure and sudden loss of consciousness. Endotracheal intubation was repeated and respiratory support was provided for another 2 days, after which she was discharged from the ICU with continuous dialysis. She was weaned off hemodialysis on day 92 but still required still required medical support even after surgical correction. She did not develop any complications associated with right heart failure, based on telephone follow-up conducted on day 300 after surgery.

## 4 Case 3

A 59-year-old female was admitted with palpitations and dyspnea on excision. TEE revealed the presence of Ebstein anomaly of the tricuspid valve with apical displacement, severe tricuspid regurgitation, PFO, bidirectional atrial shunts, mild mitral regurgitation, substantial enlargement of the right atrium, an atrialized right ventricle and left atrium, and a small left ventricle with normal systolic function. Electrocardiography showed atrial fibrillation and complete right bundle branch block.

Anesthesia was performed routinely, together with intravenous administration of methylprednisolone (80 mg). At 20 minutes before surgery, the patient experienced an episode of ventricular tachycardia, during which the heart rate rose to 180 to 200 bpm and blood pressure fell to 65/45 mm Hg. This condition resolved with amiodarone (150 mg), and hemodynamics remained stable with epinephrine (0.02 mcg/kg/min) and milrinone (0.5 mcg/kg/min).

CPB was performed similarly as for Case 2. Priming was carried out using gelofusine (1250 mL), and 20% mannitol (250 mL), and methylprednisolone (500 mg). The aorta was clamped and cold-blood cardioplegia was administered at the aortic root once at 20 mL/kg, and then again every 10 minutes thereafter at 5 mL/kg. After cross-clamping for 59 minutes, albumin (22.5 g) was administered to increase colloid osmotic pressure. During CPB, the patient underwent the following procedures: tricuspid valve replacement, partial atriectomy, unilateral bidirectional Glenn shunt under hypothermia, and implantation of epicardial pacemaker leads. CPB was continued for another 63 minutes after these procedures, then the patient was weaned off it using low-dose epinephrine (0.06 mcg/kg/min), milrinone (0.5 mcg/kg/min), and norepinephrine (0.09 mcg/kg/min). Hemodynamics stabilized at a heart rate of 70 to 100 bpm, blood pressure of 80–100/40–60 mm Hg, and right atrium pressure of 5 mm Hg. Systolic pressure was 30 mm Hg in both the right pulmonary artery and superior vena cava. TEE showed mild tricuspid regurgitation and anastomotic patency in the superior vena cava–right pulmonary artery.

Total CPB time was 132 minutes. During bypass, 1% sevoflurane was administered by inhalation from an oxygenator, and mechanical ventilation was performed at low tidal volume with the following parameters: FiO_2_, 21%; tidal volume (Vt), 100 mL; and respiratory rate (RR), 12 bpm. Ultrafiltration was used to maintain Hb concentration higher than 85 g/L and thereby avoid the need for allogeneic blood transfusion.

After the patient's admission to the ICU (day 1), ventilation was initiated with the following parameters: PEEP, <5 mm Hg; Vt, 450 mL; FiO_2_, 40%; and RR, 12 bpm. She presented with a nodal rhythm, heart beat of 80 bpm, CVP of 14 to 21 cmH_2_O, and slightly low MAP of 54 mm Hg. MAP increased to 70 mm Hg with AVP (0.015 U/min). TEE showed a right atrium size of 56 mm, an ejection fraction of 69%, and compromised right ventricle function with 6-mm systolic excursion of the tricuspid annular plane. Tissue Doppler imaging showed myocardial systolic velocity of 6.9 cm/s. The patient suffered an episode of atrial flutter on day 3, which returned to sinus rhythm after infusion of amiodarone (30 mg/h).

On day 5, inotropics were progressively decreased: AVP and norepinephrine were terminated, while epinephrine was maintained at 0.02 mcg/kg/min. The patient was later extubated, and she went on to experience a few episodes of atrial flutter, which resolved under amiodarone therapy. On day 19, she was discharged with normal sinus rhythm and stable hemodynamics. Subsequent clinical follow-up was uneventful on day 200.

## 5 Discussion

**Figure 2:**
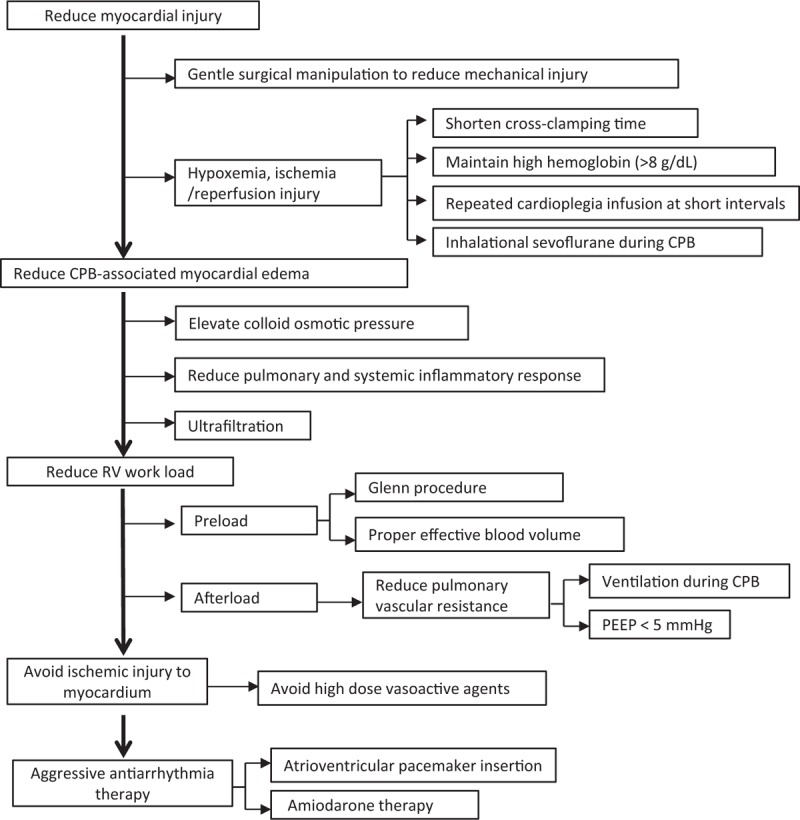
Perioperative management of patients with Ebstein anomaly to prevent right ventricle dysfunction.

Ebstein anomaly is a common congenital heart disease characterized by tricuspid valve hypoplasia with apically displaced septal and posterior leaflets, which may induce severe tricuspid regurgitation and dilation of the “atrialized” portion of the right ventricle.^[7]^ Therefore, the major pathophysiology is diminished antegrade blood flow through the right heart, decreased functional right ventricle size, and right heart dilation (Fig. 1). Myocardial contraction may become too weak to circulate blood adequately through the pulmonary circulation and into the left heart system. This condition can be exacerbated by surgically induced myocardial injury, often leading to right ventricle failure. This reduces left ventricular preload and produces a pancake effect on the left ventricle. The resulting deficiency of blood in the left heart affects systemic response to vasoactive and inotropic agents, so many patients require ECMO support. Of the 3 adults reviewed here who underwent surgery to correct Ebstein anomaly, 1 died and another experienced kidney failure despite postoperative support on ECMO. One or 2 strategies were used to prevent right ventricle failure in Cases 1 and 2, while multiple strategies were used in Case 3 (Fig. 2). Our case series suggests that appropriate perioperative treatment and protection of the myocardium can help prevent right-sided heart failure.

### 5.1 Perioperative control of atrial and ventricle arrhythmia

Patients with Ebstein anomaly have an extremely dilated right atrium and nearly half (43%) experience persistent arrhythmia, most commonly in the form of atrial flutter, atrial fibrillation, and ventricle arrhythmia.^[8]^ Such arrhythmia decreases cardiac output and increases myocardial oxygen consumption, which increases the risk of hemodynamic instability after ventricle fibrillation as well as the risk of postoperative mortality.^[9]^ Both atrial and ventricle tachycardia occurred in Cases 1 and 2. Continuous amiodarone infusion and early pacemaker insertion to override ectopic rhythm allowed reasonable control of premature heart beat.

### 5.2 Perioperative protection of myocardium

Gentle surgical manipulation can reduce mechanical injury to the myocardium and shorten the duration of surgery and myocardial ischemia. We recommend keeping surgery as brief as possible. Excessively long aortic cross-clamp time and cardioplegia interval may partly explain why Case 1 experienced postoperative right-sided heart malfunction despite good heart functional capacity at baseline.

Another factor in preserving right-sided heart functional capacity is reducing myocardial ischemia-reperfusion injury. Such injury can be reduced by keeping perfusion duration within 10 to 15 minutes,^[10,11]^ which can still allow rapid recovery of myocardial metabolism and function. For these reasons, we decided to limit cardioplegia to 10 minutes in Case 3.

Early after surgery to correct Ebstein anomaly, patients often experience postoperative myocardial edema, which can impair right-sided heart function. Such edema was the likely cause of the hypotension that occurred within 24 hours after surgery in all 3 cases in our series. To reduce inflammation-induced edema, we primed all 3 cases for CPB using mannitol. Priming of Case 3 also included colloids and albumin to increase colloid osmotic pressure. All 3 cases underwent ultrafiltration to increase colloid osmotic pressure and effective hemoglobin concentration, which can boost oxygen delivery to the right-sided heart and decrease myocardial edema.^[12,13]^ Although methylprednisolone was given in all 3 cases, it is noted that administration of methylprednisolone should not be routinely recommended based on the recent evidence.^[14,15]^ In fact, hemoglobin levels in Cases 2 and 3 were maintained higher than 8 g/dL.^[12,16]^ Postoperative hypotension in all 3 cases resolved with low-dose vasopressin, suggesting that the myocardial edema was not severe enough to compromise hemodynamics.

### 5.3 Post-CPB reduction of right-side cardiac workload

Proper circulating volume is important for preventing high preload on the right ventricle. For this reason, we monitored CVP in the right atrium as well as TEE in Case 3 when weaning her off CPB. This monitoring continued as part of bedside surveillance in the ICU.

Intentionally leaving an atrial septal defect can lower cardiac workload and oxygen consumption, which can compensate for poor function in the right side of the heart. In particular, it can increase LV preload, which can compensate for inadequate left ventricle filling during RV failure and simultaneously may decrease preload on the right ventricle. However, this approach in Case 1 did not appear to substantially reduce preload on the right ventricle, because no right-to-left shunting was observed during postoperative failure of the right-sided heart. Case 3 received a unilateral bidirectional Glenn shunt, which may have reduced preload on the right ventricle and thereby helped preserve residual function. The Glenn procedure can be a useful adjunct treatment during repair of advanced Ebstein anomaly involving severe right ventricle dilation and dysfunction,^[3–5]^ especially in patients who cannot be weaned off CPB.

Pulmonary vascular resistance significantly affects right ventricle afterload. Pulmonary atelectasis and impaired gas exchange can cause hypoxia, leading to pulmonary vasoconstriction and increased vascular resistance. Inflammation can exacerbate pulmonary vasoconstriction. Therefore, low-tidal volume ventilation was continuously applied during CPB, particularly in Case 3, to attenuate acute pulmonary injury and improve oxygenation following pulmonary vasoconstriction.^[17,18]^ Adequate PEEP was maintained during the postoperative period to avoid lung collapse, reduce atelectasis, and improve gas exchange. One problem with positive-pressure ventilation is that the resulting elevated intrathoracic pressure impairs contractibility and reduces cardiac output,^[19]^ thereby compromising right ventricle function. This makes correct selection of the PEEP value important, yet optimal values are controversial. We chose to maintain PEEP within 5 mm Hg in all 3 cases.

### 5.4 Right ventricle support

Vasoactive and inotropic agents are usually administered to support poor right ventricle contractility, and we used them in all 3 cases in our series. Epinephrine and norepinephrine are alpha receptor agonists. They can cause pulmonary artery contraction, which may aggravate right ventricle afterload, and lead to vasoconstriction in vital organs, ultimately causing multiorgan malfunction. This may explain why renal failure occurred in Case 2. In Case 3, we restricted doses of epinephrine and norepinephrine to 0.10 mcg/kg/min and of AVP to 0.035 U/min,^[20]^ since higher AVP doses have been shown to cause life-threatening myocardial ischemia and sudden cardiac death in clinical and animal studies, potentially as a result of coronary vasoconstriction.^[21,22]^ When higher doses of these agents are required, ECMO should be considered to maintain hemodynamic stability.^[3,5]^

Despite the use of ECMO, Cases 1 and 2 suffered from severe pulmonary malfunction, and Case 1 died. Case 1 showed stable hemodynamics and respiratory function as well as obvious recovery of right-sided heart functional capacity after 3 days on ECMO. Unfortunately, after the patient was weaned off ECMO, she experienced further deterioration of pulmonary function followed by hypoxemia, which may have been due to the prolonged ECMO duration. We did not perform the Glenn procedure in this patient since her impaired lung function meant that oxygenation would not be satisfactory even if pulmonary blood was abundant. Instead, we provided ECMO assistance until pulmonary function recovered.

In conclusion, the case series described here is consistent with the idea that adults with Ebstein anomaly undergoing tricuspid valve replacement or repair with or without a concomitant Glenn procedure are at high risk of postoperative right-sided heart failure. Surgical prognosis can be improved through aggressive preoperative treatment, vasoactive and anti-arrhythmia medications, and comprehensive measures designed to reduce myocardial injury, prevent myocardial edema, and reduce pre- and afterload on the right ventricle.
